# Rewiring innate and adaptive immunity with TLR9 agonist to treat osteosarcoma

**DOI:** 10.1186/s13046-023-02731-z

**Published:** 2023-06-26

**Authors:** Caterina Cascini, Chiara Ratti, Laura Botti, Beatrice Parma, Valeria Cancila, Adriana Salvaggio, Cristina Meazza, Claudio Tripodo, Mario P. Colombo, Claudia Chiodoni

**Affiliations:** 1 Department of Experimental Oncology, Molecular Immunology Unit, Fondazione IRCCS Istituto Nazionale Dei Tumori, Via Amadeo 42, 20133 Milan, Italy; 2 Department of Health Science, Tumor Immunology Unit, University of Palermo School of Medicine, Palermo, Italy; 3 Pediatric Oncology, Fondazione IRCCS Istituto Nazionale Dei Tumori, Milan, Italy; 4 IFOM, FIRC Institute of Molecular Oncology, Milan, Italy

**Keywords:** Osteosarcoma, Immunomodulation, Lymphocyte activation, Macrophages, TLR9, Mouse models

## Abstract

**Background:**

Osteosarcoma (OS) is the most common primary bone tumor in children and adolescent. Surgery and multidrug chemotherapy are the standard of treatment achieving 60–70% of event-free survival for localized disease at diagnosis. However, for metastatic disease, the prognosis is dismal. Exploiting immune system activation in the setting of such unfavorable mesenchymal tumors represents a new therapeutic challenge.

**Methods:**

In immune competent OS mouse models bearing two contralateral lesions, we tested the efficacy of intralesional administration of a TLR9 agonist against the treated and not treated contralateral lesion evaluating abscopal effect. Multiparametric flow cytometry was used to evaluate changes of the tumor immune microenviroment. Experiments in immune-deficient mice allowed the investigation of the role of adaptive T cells in TLR9 agonist effects, while T cell receptor sequencing was used to assess the expansion of specific T cell clones.

**Results:**

TLR9 agonist strongly impaired the growth of locally-treated tumors and its therapeutic effect also extended to the contralateral, untreated lesion. Multiparametric flow cytometry showed conspicuous changes in the immune landscape of the OS immune microenvironment upon TLR9 engagement, involving a reduction in M2-like macrophages, paralleled by increased infiltration of dendritic cells and activated CD8 T cells in both lesions. Remarkably, CD8 T cells were needed for the induction of the abscopal effect, whereas they were not strictly necessary for halting the growth of the treated lesion. T cell receptor (TCR) sequencing of tumor infiltrating CD8 T cells showed the expansion of specific TCR clones in the treated tumors and, remarkably, their selected representation in the contralateral untreated lesions, providing the first evidence of the rewiring of tumor-associated T cell clonal architectures.

**Conclusions:**

Overall these data indicate that the TLR9 agonist acts as an in situ anti-tumor vaccine, activating an innate immune response sufficient to suppress local tumor growth while inducing a systemic adaptive immunity with selective expansion of CD8 T cell clones, which are needed for the abscopal effect.

**Graphical Abstract:**

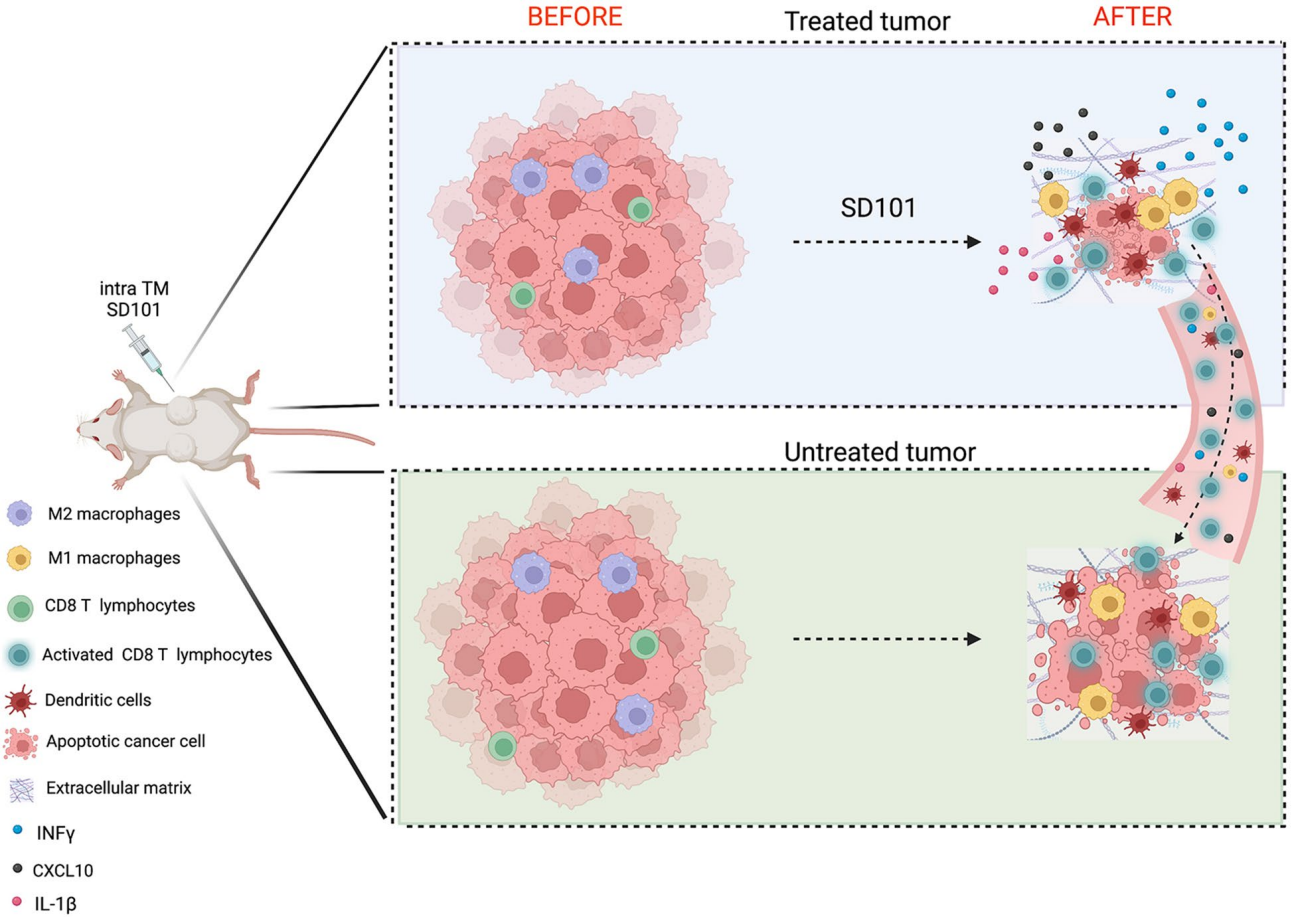

**Supplementary Information:**

The online version contains supplementary material available at 10.1186/s13046-023-02731-z.

## Background

Osteosarcoma (OS) is a high-grade malignant stromal tumor composed of mesenchymal cells producing osteoid and immature bone with a peak of incidence in the second decade of life [1]. Although relatively rare, the social impact of this neoplasm is particularly relevant. In contrast to carcinomas, the molecular genetics of osteosarcoma progression and the role of its particular tumor microenvironment (TME), the bones, remain largely unknown [2]. Indeed, while the TME has been widely studied in other solid tumors to describe its role in tumor progression and, in part, in distant dissemination [3], the investigation of tumor-stroma interaction in OS has been quite neglected for decades, in part because of the rarity of the neoplasia, of the peculiar site of OS development in bones, and of the paucity of syngeneic mouse models that allow to study the involvement of both innate and adaptive immune cells. However, thanks to the recent technological advance in transcriptomics, proteomics and bioinformatics, a new wave of knowledge on OS TME has been acquired. The general view is that OS is a cold tumor, with a scant immune infiltrate, composed mainly of macrophages and other myeloid cells with very few T cells. As for other tumor types, most studies indicate that T cell infiltration correlates with a good prognosis and better overall survival [4, 5]. Still, some of the available data are somewhat contradictory when compared to other cancer types. For example, while the pro-tumorigenic role of tumor-associated macrophages (TAMs) has been widely demonstrated in several carcinomas and their poor prognostic value has been established in a large fraction of tumors, the role of TAMs in OS has not yet been clearly defined, yet. Some pieces of evidence support the role of TAMs, either M1 or M2, in the suppression of metastasis in high grade OS, [6] while other studies indicate their pro-tumoral role and correlation with poor prognosis [7–9]. This unclear scenario requires further investigation. As the tumor microenvironment in its cellular and/or molecular components may represent new therapeutic targets, correct evaluation of its composition and knowledge of its role in malignant progression have become critical. This is particularly relevant for OS, for which no biological/targeted therapies are available. Indeed, bone sarcomas therapy is still firmly entrenched in conventional cytotoxic drugs [10] and state of the art therapy comprises neo-adjuvant and adjuvant multidrug chemotherapy before and after surgery, respectively. Today, the patients can expect a 60–70% chance of achieving a complete remission in presence of a localized tumor at diagnosis. However, the prognosis of patients with metastasis remains poor and few treatment options can be offered to patients who relapse after first line therapies.

Immune checkpoint inhibitors, such as anti PD-1 or PD-L1 antibodies, have been designed to target negative regulatory pathways of T cell activity, to boost or restore their anti-tumor function, and they have indeed been shown to achieve considerable clinical results, at least in specific tumor types, such as melanoma and lung cancer [11–13]. Regretfully, immune checkpoint inhibitors have not shown convincing clinical results in sarcomas so far, and the expression and role of the PD-1/PD-L1 axis have not been clearly defined yet [14, 15]. One possible reason for such a failure is the paucity of infiltrating T cells and the immune suppressive characteristics of their TME, suggesting that other immune-modulatory drugs could be more effective for increasing immune cell infiltration and revert immune suppression, thus rendering tumors more susceptible to immune checkpoint inhibitors.

The Toll-like receptors (TLRs) are sensors of pathogen associated molecular patterns (PAMPs) that allow recognition of pathogens and protect the host from pathogen infection. In particular, TLR9 represents a link between innate and adaptive immunity. When its ligand, a CpG-ODN, is provided, antigen-presenting cells (APC) such as DCs and macrophages are activated to generate an innate immune response, which is then followed by the initiation of an adaptive response, with recruitment and activation of lymphocytes. In light of these immune stimulatory features, here we tested the therapeutic efficacy of a TLR9 agonist, administered intra-lesion, in immune-competent mouse models of OS demonstrating its activity not only on the treated lesion but also on a contralateral, untreated, tumor, suggesting the activation of both innate and adaptive immune response capable of inducing an abscopal effect.

## Methods

### Mice

Female 8-week-old BALB/c and nu/nu mice, were purchased from Charles River Laboratories (Calco). Mice were maintained in the Animal Facility of Fondazione IRCCS Istituto Nazionale dei Tumori. Animal experiments were approved by the Institute Ethical Committee and Italian Ministry of Health (project INT 06_2020, authorization #1010/2020-PR) and were performed in accordance with national law (D.lgs 26/2014).

### Cell lines

The osteosarcoma mOS69 cell line was established in our laboratory, as previously described [16]. The K7M2 OS cell line was purchased from LGC Standards (ATCC, CRL#2836). Cells were grown in a humidified incubator at 37 °C with 5% CO_2_ and maintained in standard medium, Dulbecco's modified Eagle’s medium with 10% FBS. The cell lines were checked for Mycoplasma infection every 2–4 months by PCR Mycoplasma Detection Kit (PanReac Applichem).

### In vivo experiments

Twelve-week old female BALB/c and CD1-nude (nu/nu) mice were purchased from Charles River (Calco, Italy). Tumor cells were injected subcutaneously, in one or both flanks, at a dose of 2 × 10^5^ and 10^6^ cells, for mOS69 and K7M2 lines, respectively. Tumor mass was measured with a caliper and tumor volume (mm^3^) was calculated [long diameter × (short diameter)^2^/2]. The sequence of the TLR9 agonist SD101 was derived from Marshall et al. [17] and was synthetized by Merck (Sigma Aldrich). For in vivo experiments, SD101 was injected intratumorally at a dose of 25–50 μg twice a week, for a total of four doses, starting when tumors reached 4–5 mm in diameter. Anti-mouse PD-1 (RMP1-14 clone, InvivoMab) or control antibody (rat IgG2a Isotype Control) was administered intraperitoneally (i.p.) (200 μg/mouse) twice a week, starting 24 h after the first SD101 injection, for a total of four injections. All antibodies used in vivo were purchased from BioXCell.

For macrophage depletion, when tumors reached 4–5 mm in diameter, mice were treated three times a week with clodronate, or control liposomes, intra-tumor (50μl/mouse), for 3 weeks. Clodronate liposomes and control liposomes were purchased from Liposoma BV (Amsterdam, The Netherlands).

To evaluate the effect of SD101 on metastatic potential, 2 × 10^5^ mOS69 cells were injected intra-vein (i.v.) and 10 days after tumor cell injection intranasal SD101 treatment (10 μg/50 μl in saline) or saline control treatment was performed thrice a week for a total of six doses. An intranasal dose volume of 50 μl was chosen to maximize exposure to the lungs and mimic inhalation in mice [18]. Lung metastases were counted blindly by two operators through the histological evaluation on serial lung sections (two sections for each sample), stained with hematoxylin and eosin (H&E). The metastatic area was quantified using the Leica Las Core software under a DM4B Leica microscope. The percentage of lung area occupied by metastases was measured as follows: (sum of areas of all metastatic lesions /total lung area) × 100. For each in vivo experiments 5–6 animals per group are used. Specific number of mice is reported in each figure legend.

### Histology, immunohistochemistry and immunofluorescence

Tumors were excised, washed in PBS, fixed in 10% neutral buffered formalin, and paraffin embedded. Four-micrometers-thick mouse tissue sections were deparaffinized, rehydrated and H&E stained for tumor histotype definition.

Sections were unmasked using Novocastra Epitope Retrieval Solutions pH6 or pH9 in thermostatic bath at 98 °C for 30 min and after neutralization of the endogenous peroxidase with 3% H_2_O_2_ and Fc blocking by 0.4% casein in PBS (Novocastra), and incubated with antibodies over night at 4 °C (Additional file 1 for list of antibodies). For multiple-marker immunostaining, sections were subjected to sequential rounds of single-marker immunostaining, and the binding of the primary antibodies was revealed using specific secondary antibodies conjugated with different fluorophore. IHC staining was developed using the IgG (H&L)-specific secondary antibodies (Life Technologies, 1:500) or ImmPRESS-AP Goat anti-Rat Polymer Detection Kit (cod. MP-5444–15, Vector Laboratories) and DAB (3,30-Diaminobenzidine, Novocastra) or Vulcan Fast Red (BioOptica) as substrate chromogen. Anti-mouse and anti-rabbit secondary antibodies (Alexa Fluor 488 and 568 conjugate) were used for immunofluorescence. Collagen deposition was visualized using Masson’s Trichrome Stain (Cod. 010210, Diapath) following manufacturer’s instructions.

The slides were analyzed under a Zeiss Axioscope A1 microscope equipped with four fluorescence channels with widefield IF. Microphotographs were collected using a Zeiss Axiocam 503 color digital camera with the Zen 2.0 Software (Zeiss).

### Flow cytometry

Tumor sample leukocyte infiltration was evaluated by flow cytometry analysis as described previously [16], using antibodies for the different immune cell subsets (Additional file 1). For T regulatory cell detection, after CD4 surface staining, cells were fixed, permeabilized, and stained with FoxP3 antibody, following the manufacturer’s instruction (eBioscience). Samples acquired using a BD LSR II Fortessa instrument were analyzed using the FlowJo software (TreeStar).

### RNA extraction and quantitative (q)PCR

Tumor samples collected and stored in RNAlater solution (Thermo Fisher) were mechanically disrupted using TRIzol reagent (Invitrogen). RNA was purified by phenol/chloroform extraction and then loaded onto RNeasy MINI or MICRO kits (Qiagen) with on-column DNAse treatment. RNA purity and yield were assessed using NanoDrop spectrophotometer. RNA was reverse transcribed using the High Capacity cDNA Reverse Transcription Kit (Thermo Fisher). The TaqMan® Array Mouse Immune Panel (Thermo Fisher) was used to evaluate the tumor immune landscape following the manufacturer’s instruction.

### TCR sequencing

Genomic DNA was extracted from the tumor samples using the QIAamp DNA Mini Kit (QIAGEN) following the manufacturer’s instructions. DNA quality and quantity were assessed using the Qubit fluorometer (Invitrogen). Three micrograms of genomic DNA were used for TCR sequencing, performed by ImmunoSEQ Service using the immunoSEQ Mouse TCRB Assay (Adaptive Biotech, Seattle, WA, USA). Data were analyzed using the ImmunoSEQ Analyzer software.

### Statistical analysis

Tumor volume data are represented as mean ± SD with Student’s unpaired 2-tailed t-test or Mann Whitney U-test used for statistical analysis. Flow cytometry data are shown singularly asmean ± SD, and were analyzed by Mann Whitney U-test. PCR data from TaqMan® array mouse immune panel experiments were analyzed by ANOVA (Kruskal–Wallis test) using Prism 9 software (GraphPad).

For lung metastasis evaluation, data are represented singularly with graphs showing the median; statistical significance was evaluated with Mann Whitney U-tests using Prism 9 software.

## Results

### Intratumoral administration of a TLR9 agonist exerts an abscopal effect on the contralateral, untreated, tumor lesion

**Fig. 1:**
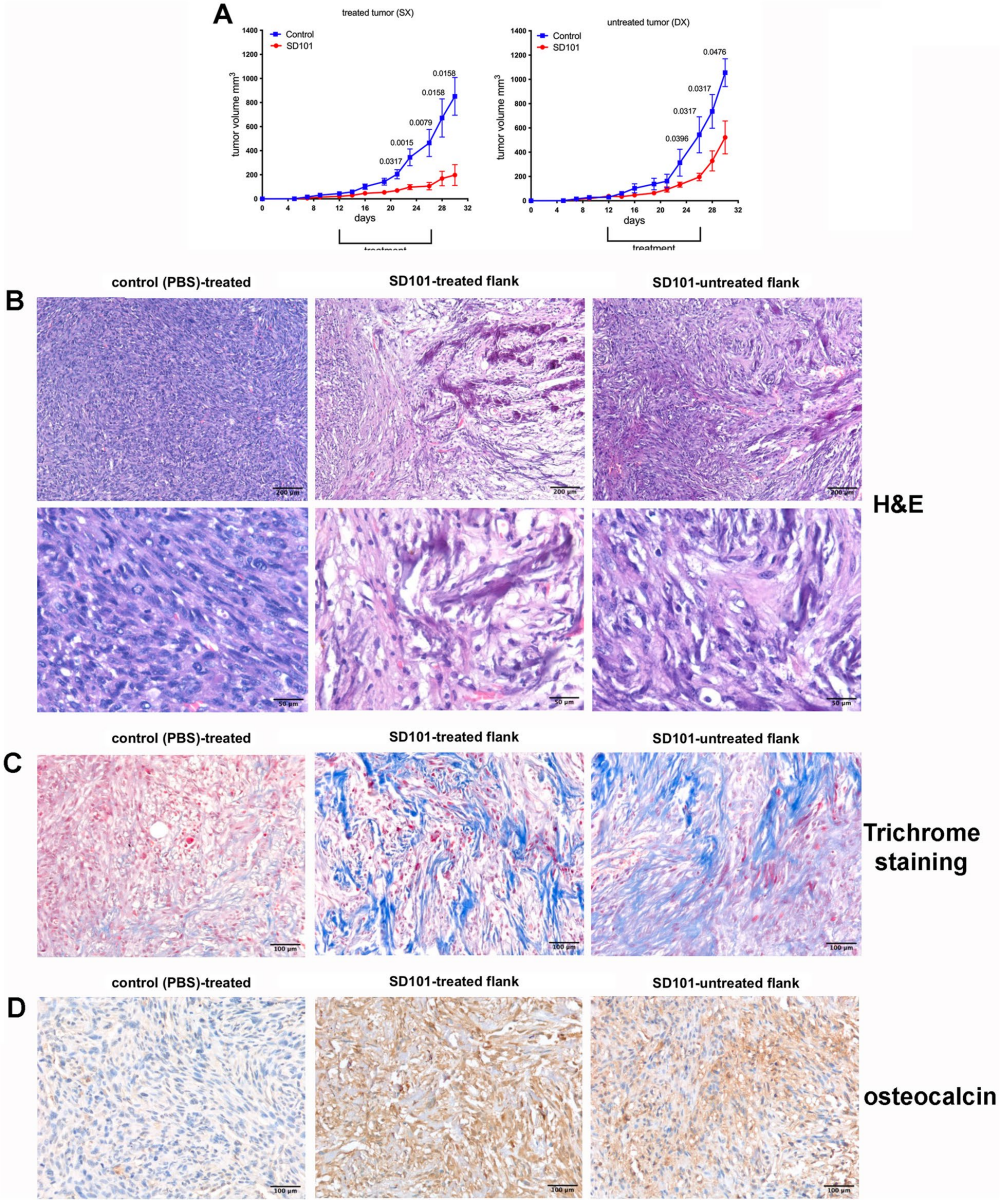
Intratumoral administration of TLR9 agonist in osteosarcoma tumors shows therapeutic efficacy on both treated and distant lesions. **A**. Graphs show mean volume of mOS69 tumors SD101-treated (left panel) and untreated (right panel). Five animals per group were used. A representative experiment is shown (out of 4 experiments performed). Multiple unpaired t tests, one per time points, were used for statistical analysis. **B**. Representive histological analysis of tumor lesions in Hematoxylin & eosin. **C**. Representative images of Masson’s Trichrome staining showing a significant increase in collagen (blue staining). **D**. Representative images of osteocalcin immunohistochemistry, suggestive of increased tumor cell differentiation and matrix deposition

To assess the therapeutic efficacy of the TLR9 agonist SD101 in an immunocompetent osteosarcoma mouse model (mOS69), tumor cells were injected subcutaneously into both flanks and only one lesion was treated. This system allows evaluation of the abscopal effect on the contralateral, untreated, tumor mass. The treatment almost completely eradicated the treated lesions and severely impaired the growth of the contralateral tumors (Fig. 1A, right panel and Additional file 2). Histopathological evaluation of the tumors indicated a lower histological grade in SD101-treated lesions, with fewer neoplastic cells, increased osteoid matrix deposition and conspicuous inflammatory infiltrate compared to PBS-treated controls, which showed high-grade tumors with almost no matrix deposition and very few immune infiltrating cells (Fig. 1B). The induction of matrix deposition and tumor cell differentiation was confirmed by Masson’s Trichrome staining and immunohistochemistry for osteocalcin (Fig. 1C and D). Interestingly, the contralateral, untreated, lesion in mice receiving SD101 into the opposite tumor showed an intermediate phenotype, with some inflammatory infiltrate and partial osteoid matrix deposition.

To assess whether TLR9 had any direct effect on neoplastic cells, we treated mOS69 cells in vitro with different doses of SD101 for 24 and 48 h and tested its effect on proliferation and apoptosis induction. As shown in Additional file 2 (Figure S1B-C), we did not observe any significant modulation of either proliferation (ki67 expression) or apoptosis (annexinV/7AAD staining).

### Intralesional injection of a TLR9 agonist reprograms myeloid cells in the tumor microenviroment

**Fig. 2:**
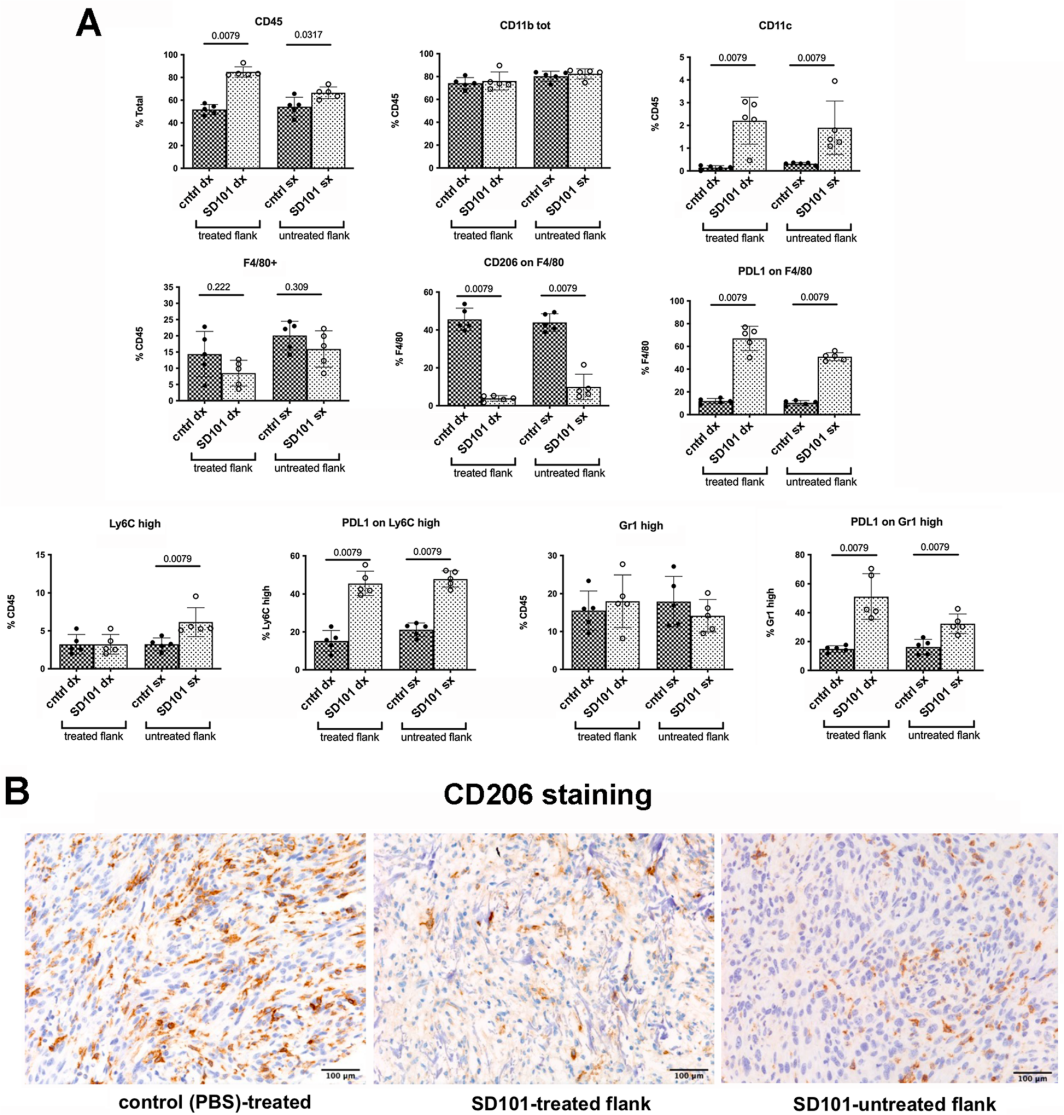
Intratumoral administration of TLR9 agonist reprograms the tumor microenvironment, decreasing the number of M2 polarized macrophages and increasing dendritic cell infiltration. **A**. Multiparametric flow cytometry analysis of tumor infiltrating myeloid cells in both treated and untreated lesions from SD101- or PBS-treated mice. Data are expressed as percentage of specific cell subsets (shown on the Y axis). Data from single mice are shown (4–5 mice per group; a representative experiment is shown). Mann–Whitney test was used for statistical analysis. **B**. Representative immunohistochemistry staining of CD206 on tumor lesions

As TLR9 is mainly expressed by cells of the immune system, we assessed whether SD101 local administration altered the immune tumor microenvironment of both treated and controlater, untreated, lesions. In agreement with the histopathological analysis, we confirmed, by flow cytometry, an overall increased infiltration by CD45 + immune cells (Fig. 2A). Although we did not detect significant changes in the percentage of total myeloid cells (CD11b + cells), macrophages (F4/80 +), and granulocytic myeloid cells (Gr-1^high^), we observed a significant increase in dendritic cells (CD11c +) in SD101-treated mice. Interestingly, SD101 administration significantly modified the phenotype of infiltrating macrophages, with an almost complete depletion of CD206 + , M2-like, macrophages (Fig. 2A and S1D for gating strategy), which was confirmed by IHC analysis (Fig. 2B). Notably, such alterations in immune cells were detected in both treated and contralateral, untreated, lesions, suggesting the ability of the TLR9 agonist to reprogram the TME locally and at a distant site. In agreement with the ability of TLR9 to induce IFNs production, the expression of PD-L1 was increased in all infiltrating myeloid cell subsets (Fig. 2A).

### Reprogramming tumor-associated macrophages is therapeutically more efficient than their direct depletion

In light of the above results and of the controversial role of tumor-associated macrophages (TAMs) in OS aggressiveness we also investigated whether liposome-encapsulated clodronate-mediated macrophage depletion could affect OS growth as SD101 does. Intralesional clodronate treatment, while almost completely depleting TAM, as assessed by flow cytometry, was unable to impair tumor growth of treated and contralateral, untreated, lesions (Additional file 3). Residual macrophages in clodronate-treated tumors showed reduced CD206 and PD-L1 expression. Clodronate treatment also increased the number of Gr-1^high^ myeloid cells while leaving unchanged the percentage and phenotype of CD8 + T cells and decreased the percentage of CD4 + T cells, even more of the fraction of T regulatory cells (Tregs; Additional file 3).

These results suggest that the depletion of TAMs is not sufficient to impair OS growth, whereas their reprogramming toward an M1-like phenotype can change the TME and favor the recruitment and activation of CD8 T cells, as investigated below.

### TLR9 agonist turns OS microenvironment from “cold” to “hot”

**Fig. 3:**
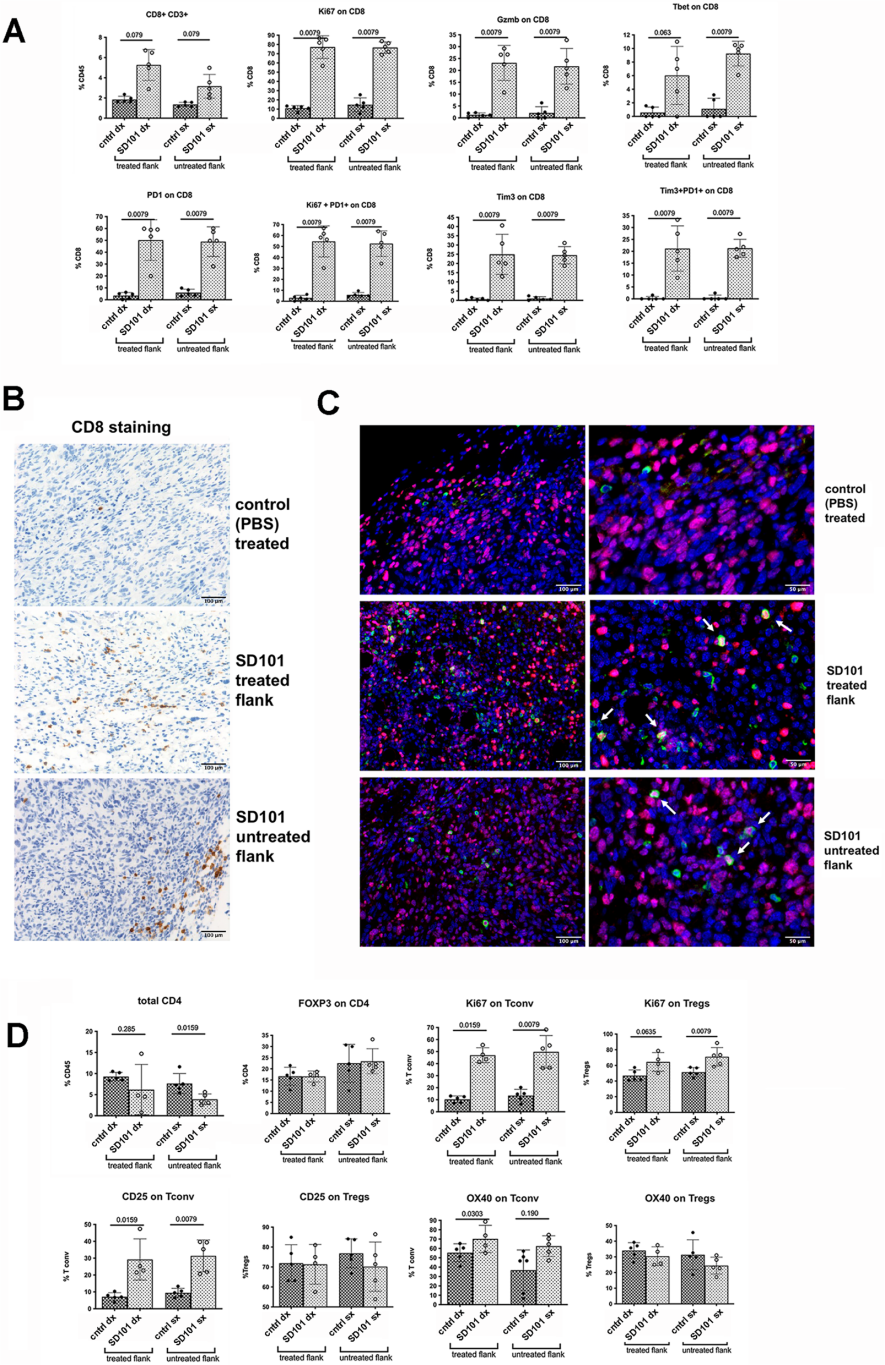
Intratumoral administration of TLR9 agonist reprograms the tumor microenvironment, with increased infiltration and activation of adaptive CD8 T cells. **A**. Multiparametric flow cytometry analysis of tumor infiltrating CD8 T cells in both treated and untreated lesions from SD101- or PBS-treated mice. Data from single mice are shown (4–5 mice per group; a representative experiment is shown). Mann–Whitney test was used for statistical analysis. **B**. Representative immunohistochemistry staining of CD8 on tumor lesions. **C**. Immunofluorescence analysis for CD8 (green) and Ki67 (pink) on tumor lesions (DAPI:blue). White arrows indicated double stained cells. Upper panel:PBS-treated control lesion; middle panel: SD101-treated lesion; lower panel: SD101-untreated lesion. **D**. Multiparametric flow cytometry analysis of tumor infiltrating CD4 T cells in both treated and untreated lesions from SD101- or PBS-treated mice. Data from single mice are shown (4–5 mice per group; a representative experiment is shown). Mann–Whitney test was used for statistical analysis

In light of the therapeutic efficacy of local TLR9 administration on contralateral untreated tumors, we investigated the effect of the treatment on the adaptive immune compartment that could be responsible for the systemic, abscopal, effect. In mice treated with SD101, the number of CD8 + CD3 + T cells increased significantly along with their proliferation (Ki67 + cells) and activation (PD1 + Ki67 + cells). Additionally, infiltrating CD8 T cells showed a much higher expression of T-bet and granzyme B, two markers of cytotoxic effector cells (Fig. 3A). Similarly to myeloid cell modifications, we observed phenotypic changes in CD8 T cells infiltrating both treated and contralateral untreated lesions. The increased number of CD8 + T cells was confirmed by IHC analysis (Fig. 3B) and their proliferation state by double IF (Fig. 3C). In contrast to CD8 + T cells, CD4 + lymphocytes were slightly reduced in SD101-treated mice, without changes in the ratio of T regulatory cells (Tregs) to conventional CD4 T cells (T conv). Notably, while T conv cells in SD101-receiving mice were highly proliferating (Ki67 +) and activated (CD25 + , OX40 +), Tregs were not (Fig. 3D).

To gain further insight into the reprogramming of the tumor microenvironment upon SD101 treatment, we analyzed the expression of several inflammation/immune-related markers in tumor samples from mice at 24 h or 7 days after the last treatment. In addition to confirming flow cytometry results, as in the case of *Cd8a, Cd3a* and *GrzmB*, the analysis showed an up-regulation of several markers involved in T cell activation and cytotoxic activity (*Cd38, Il2ra, Il7, Ifng, Prf1, Icos*), inflammation (*Il1a and Il1b*), recruitment of T cells (*Cxcl10*) and myeloid cell recruitment and activation (*Ccl2 and 3, Ccr2, Csf3, Nos2*) (Additional file 4) As expected the highest up-regulation was found in locally-treated samples 24 h after the last treatment and decreased at the 7 day-time point, whereas in contralateral, untreated, lesions it was less robust (Additional file 4).

### TLR9 agonist extends the survival of tumor-bearing mice

To further investigate the therapeutic efficacy of the TLR9 agonist, mice were challenged with tumor cells, treated with SD101 for two weeks and then left untreated to assess the effect on survival. For ethical reason, the humane endpoint was set when the tumors reached 10 mm in diameter. As shown in Additional file 5, while 100% of untreated mice were dead by day 23, 57% of SD101-treated animals were still alive at day 42 and in 14% of the mice tumor regressed completely.

### Intratumoral administration of a TLR9 agonist shows therapeutic efficacy in an additional OS tumor model

The therapeutic efficacy of SD101 was confirmed in an additional immune-competent OS tumor model, K7M2, a commercially available cell line that is widely used in the literature. Similarly to mOS69 tumors, SD101 efficiently halted the growth of treated lesions and partially controlled the contralater, untreated, tumors, although less efficiently than in the mOS69 model (Additional file 6). Multiparametric flow cytometry analysis of the treated lesion confirmed most of the changes in the immune microenvironment observed in the mOS69 model (Additional file 6). Different were the alterations in the immune compartment of contralateral, untreated, lesions, which were limited likely explaining the lower efficiency in inhibiting its growth.

### CD8 T cells are dispensable for the inhibition of primary tumor growth, but necessary for the induction of an efficient abscopal effect

**Fig. 4:**
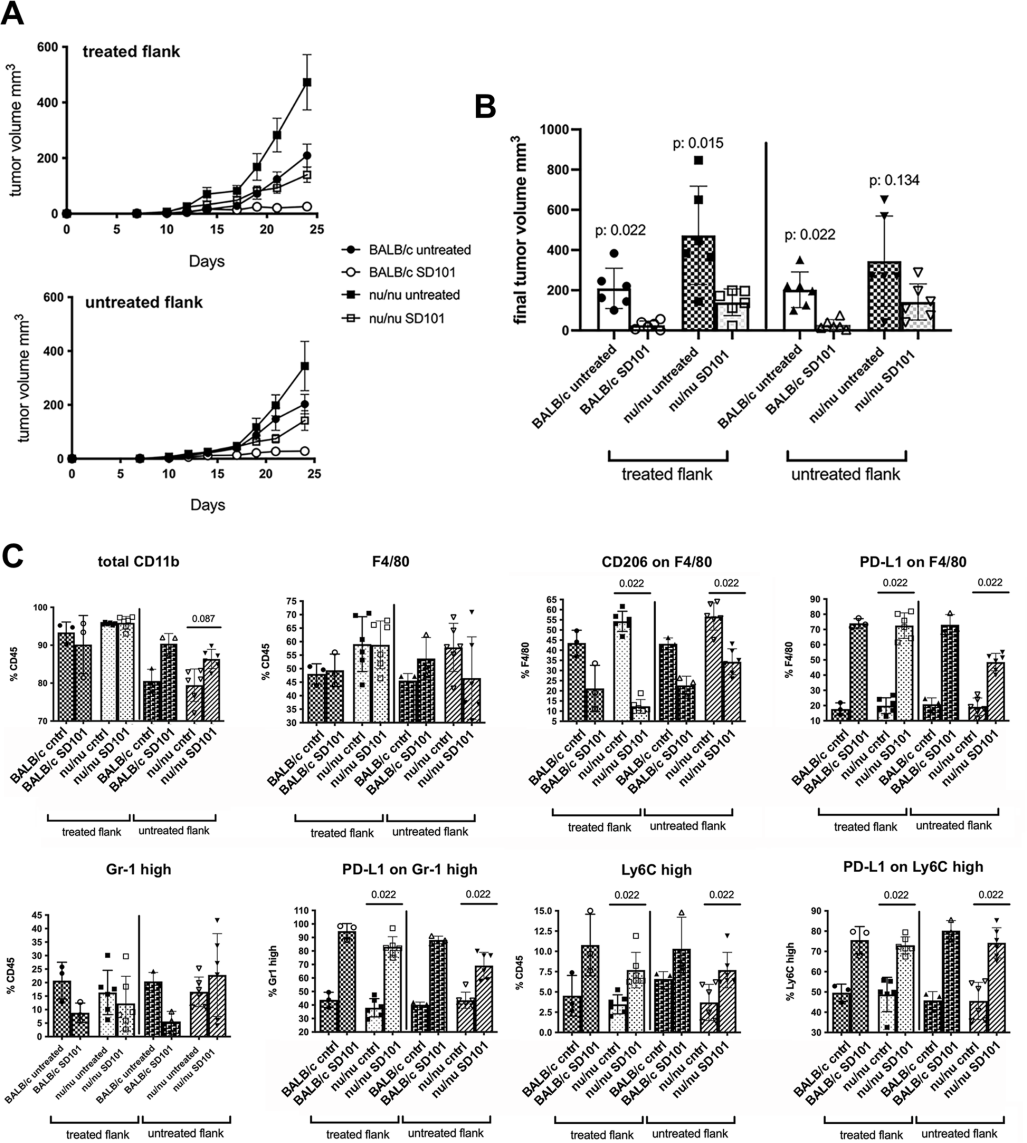
Adaptive CD8 T cells are required for therapeutic efficacy on controlater, untreated lesion. **A**. Graphs show mean volume for SD101-treated (upper panel) and untreated (lower panel) mOS69 tumors. Six animals per group were used. **B**. Graph shows the final tumor volumes from the experiment in panel A. Mann–Whitney test was used for statistical analysis. **C**. Multiparametric flow cytometry analysis of tumor infiltrating myeloid cells in both treated and untreated lesions from SD101- or PBS-treated mice. Data are expressed as percentage of specific cell subsets (shown on the Y axis). Data from single mice are shown (3 mice for BALB/c groups and 6 mice for nu/nu groups were analyzed). Mann–Whitney test was used for statistical analysis

In light of the increased number and activation of tumor infiltrating CD8 T cells in SD101-treated mice, we tested their role in treated and untreated contralateral lesions in tumor bearing mice, either immune competent (BALB/c mice) or immune-deficient (nu/nu). Although the initial tumor growth was accelerated in nude mice, SD101-treatment was as effective as in immunocompetent mice against the treated lesion but unable to significantly halt the contralateral lesion (Fig. 4A, B). To further investigate this finding, we analyzed the changes induced in the immune infiltrate in the two mouse strains by flow cytometry and found very similar changes in all myeloid cell subsets, including the striking reduction in CD206 + macrophages, with no difference in the total macrophage percentage, and in the increased expression of PD-L1 in the different subsets (Fig. 4C). This result suggests that reprogramming of the myeloid subsets is sufficient to halt tumor growth locally in the treated lesions, but unable to significantly impair the outgrowth of distant, untreated, tumors.

### T cell receptor sequencing of tumor infiltrating CD8 T cells indicated the expansion of specific clones in mice receiving TLR9 agonist

**Fig. 5:**
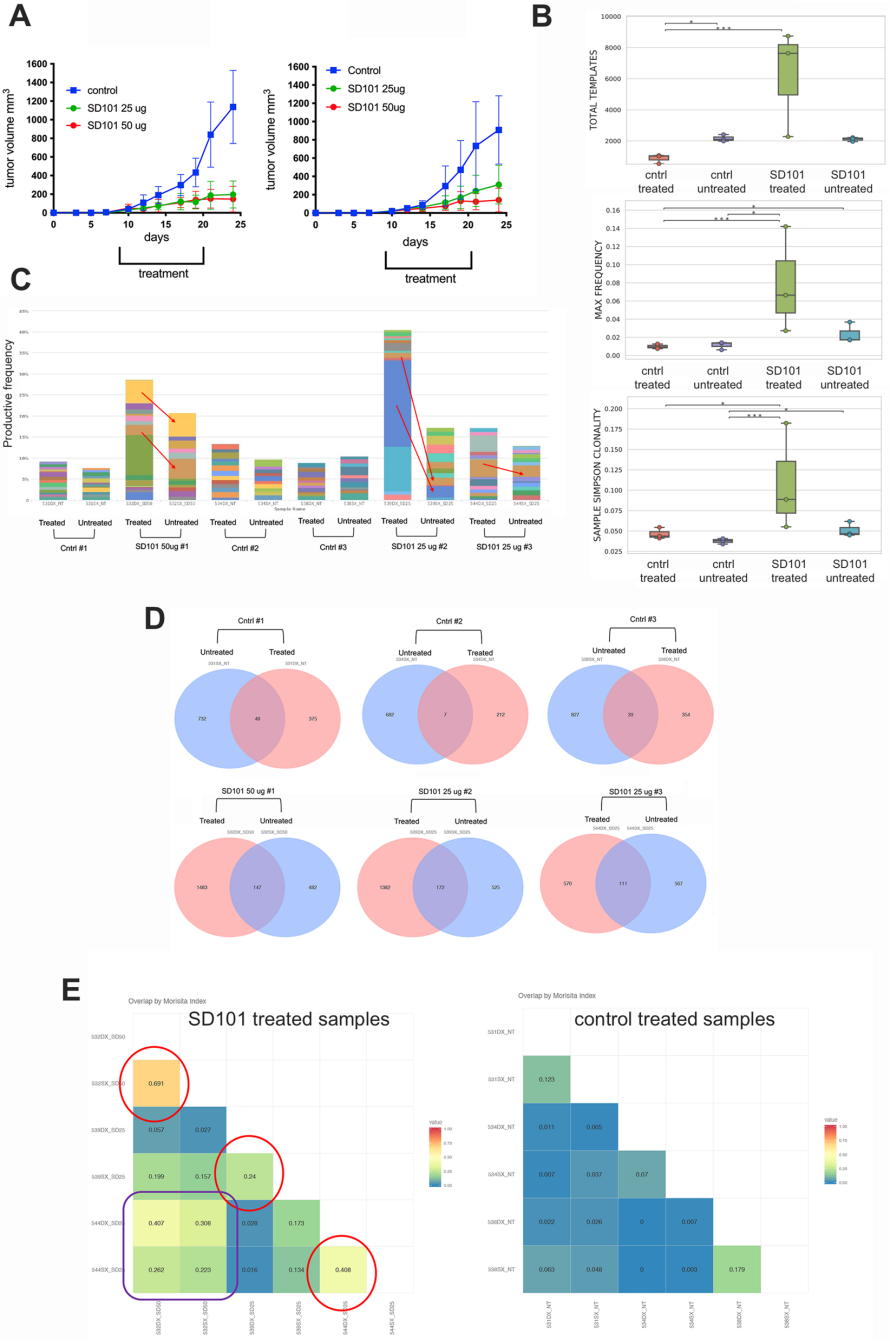
Intratumoral administration of TLR9 agonist induces the expansion of specific CD8 T cell clones in both treated and distant lesions. **A**. Graphs show mean mOS69 tumor volume for SD101-treated (left panel) and untreated (right panel) lesion. Five animals per group were used. **B**-**E**. TCR sequencing analysis has been performed on three control samples (both PBS-treated and untreated) and 3 SD101-treated samples (two treated with 25 μg and one with 50 μg, both treated and untreated lesions). Data were analyzed using the ImmunoSEQ Analyzer software. **B**. Graphs showing total templates (upper panel), max frequency (middle panel) and sample Simpson clonality (lower panel) in each group (control-treated, control-untreated, SD101-treated, SD101-untreated). **C**. Graph showing the productive frequency of each TCR clone in each samples. Each color represents a specific TCR clone. **D**. Venn diagrams showing for each mouse the clones shared between treated and untreated lesion. **E**. Overlap analysis by Morisita index showing overlapping clones between samples from the same mouse or shared between samples from different mice in SD101-treated mice (left panel) and control-treated mice (right panel)

To further investigate the role of CD8 + T cells in the therapeutic efficacy of the local administration of the TLR9 agonist, we performed the T cell receptor (TCR) sequencing of T cells infiltrating both SD101- and PBS-treated and untreated tumors. To this end, we have treated tumor-bearing mice with two different doses of the TLR9 agonist, 25 and 50 μg, to assess also whether there was a dose-dependent effect. We did not observe any difference in the inhibition of the treated lesion with both doses, whereas the efficacy against the contralateral, untreated, tumor was slightly better with the higher dose (Fig. 5A). Genomic DNA was extracted from both treated and untreated tumors from three PBS-treated and three SD101-treated mice (two animals receiving 25 μg and one receiving 50 μg). We firstly analyzed TCR sequencing results comparing the four sample groups, that is, treated and untreated tumor samples from control (PBS)-injected mice and treated and untreated samples from SD101-injected mice. As shown in Fig. 4B (upper panel) the number of total templates, which included all productive and unproductive rearrangements in the samples, was significantly higher in SD101-treated samples than in the three other groups (productive rearrangements are unique rearrangements in a sample that are in-frame and do not contain a stop codon and therefore can produce a functional protein receptor). Moreover, the maximum productive frequency (i.e. the frequency of a specific productive rearrangement among all productive rearrangements within a sample) in both treated and untreated tumor samples from mice receiving SD101 was significantly higher than that in both treated and untreated tumor samples from control PBS-treated animals (Fig. 5B, middle panel). Similar results were observed for Simpson Clonality, which measures of how much of the repertoire is made up of expanded TCR clones (Fig. 5B, lower panel). The comparison of the treated with the contralateral, untreated, lesion within the same mice clearly showed that in mice receiving SD101, TCR clones activated and expanded in the treated tumors, were also present in the untreated lesion, suggesting that tumor-specific CD8 T cell clones can likely home to the draining lymph nodes and then recirculate reaching the secondary tumor site (Fig. 5C, D). Overlap analysis by Morisita index confirmed that the majoritiy of overlapping clones were found in samples from the same mice and additionally showed that some clones may also be shared between samples from different mice (Fig. 5E, left panel). No significant overlap was detected in samples from control mice, either within the same mice or from different animals (Fig. 5E, right panel).

### Combination with immune checkpoint blockade reduces metastatic dissemination

**Fig. 6:**
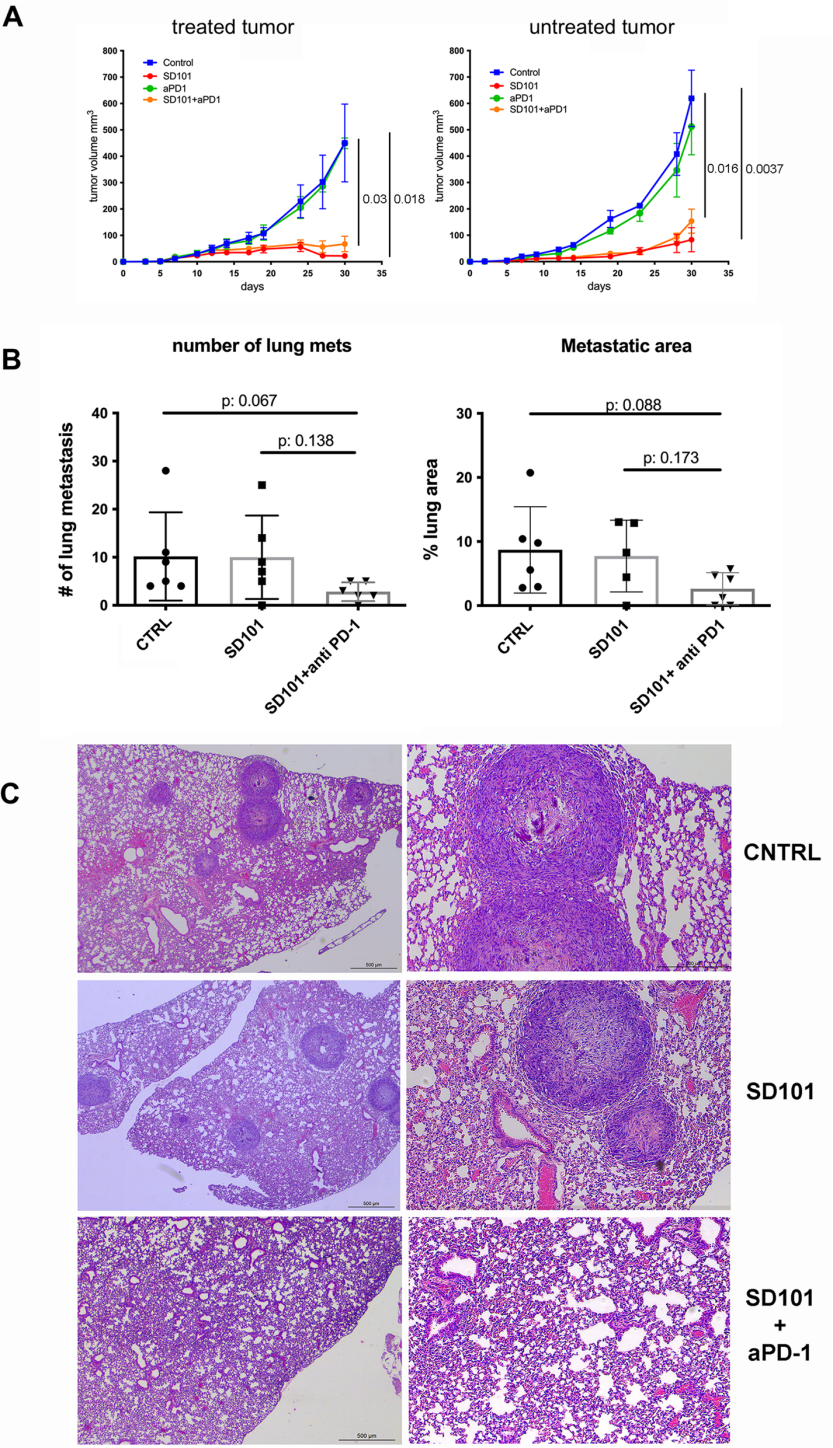
Combination treatment with anti PD-1 antibody does not increase SD101 control of primary tumor growth, but decrease metastatic dissemination. **A**. Graphs show mean mOS69 tumor volume for treated (left panel) and untreated (right panel) lesions. Five to seven animals per group were used. A representative experiment is shown (out of 3 experiments performed). Multiple unpaired t tests, one per time points, were used for statistical analysis (on graphs only the last time point p values are shown). **B**. Graphs show the number (left panel) and lung area of lung metastases (righ panel) of mOS69 injected i.v. Lung metastases were counted by histological evaluation on serial lung Sects. (2 sections for each sample), stained with hematoxylin and eosin (H&E) in blind by two operators and metastatic area quantified using Leica Las Core software under a DM4B Leica microscope. Percentage of lung area occupied by metastases was measured as follows: (sum of areas of all metastatic lesions /total lung area) × 100. six animals per group were used. **C**. Representative histological images of lungs from control, SD101-treated and SD101 + anti-PD-1 receiving animals

In light of the increased expression of PD-1 on tumor-infiltrating CD8 T cells and of PD-L1 on myeloid cells, we tested whether the combination of the TLR9 agonist with systemic administration of anti-PD-1 antibody would increase its therapeutic efficacy. However, no difference was detected when SD101 was used alone or in combination with the immune checkpoint inhibitor (Fig. 6A) both in the locally treated lesion and in the contralateral, untreated tumors. As we have previously shown [16], anti-PD-1 monotherapy did not exert any anti-tumor activity in this OS model.

In contrast, in the experimental lung metastatic setting in which tumor cells were directly injected i.v. and SD101 was administered by intranasal instillation to reach the lungs, the combination with anti PD-1 antibody was effective in reducing the metastatic load (in terms of both number of metastases and lung area occupied by metastases), whereas SD101 alone did not show any therapeutic effect (Figure 6B-D).

## Discussion

We provide evidence that intratumoral administration of SD101, a type C CpG oligonucleotide triggering TLR9, into mouse osteosarcoma models induces a massive reprogramming of the tumor microenvironment (TME), sufficient to halt the growth of treated and also of contralateral untreated lesions (graphical abstract). In addition to such boost in immune cell infiltration, neoplastic cells become more differentiated and osteoid matrix deposition is increased. Such “side effect” on tumor cell differentiation most likely contributes to the therapeutic efficacy of the treatment.

Other studies in preclinical models have shown that intratumoral administration of agents that can trigger an innate immune response, in turn can stimulate both local and systemic antitumor responses [19, 20]. The concept of the “abscopal effect”, that is, the regression of a tumor lesion outside the treatment field or even distant metastasis, has been mainly applied to explain the distant effect of local radiotherapy and then extended to other therapeutic approaches, including immune-modulatory agents such as TLR agonists, administered intralesional.

Although systemic administration is the conventional approach for anti-tumor drug delivery, it also has several limitations, such as poor delivery and penetration at the tumor site and systemic toxicities, such as auto-immune-like reactions and inflammation. Local, intra-tumor administration on the other hand may achieve a higher drug concentration at the tumor site and avoid systemic inflammation, which is particularly relevant when using immuno-stimulatory agents, such as TLR agonists.

The potency of TLR9 agonists in enhancing the anti-tumor response has been reported in several preclinical studies since the early 2000s [21]. Their local administration, either as monotherapy or in combination with radiation, chemotherapy or immunotherapy has proven efficacious in halting tumor growth likely through a consistent reprogramming of the immune TME [22–24]. TLR9 is expressed in the endosomes of immune cells, mostly of the innate compartment, such as macrophages and plasmacytoid dendritic cells, but also in other antigen presenting cells, such as B lymphocytes. The recruitment and/or reprogramming of myeloid cells toward an anti-tumor phenotype, in turn, favors the (re-)activation of adaptive T cells and in some settings improve the response to immune checkpoint blockade [25]. Similarly, in the 4T1 mammary tumor model, spontaneously metastatic to the lungs, the intra-nasal injection of the TLR9 agonist SD101 causes profound remodeling of the lung microenviroment with dendritic cell expansion and the formation of tertiary lymphoid structures adjacent to lung tumors [26]. In our study we similarly demonstrated a complete rewiring of the immune TME. Indeed in SD101-receiving mice, we observed an overall increase in immune cells infiltration, with a consistent reduction in M2-like macrophages, paralleled by an increase in CD11c + dendritic cells. Such modifications in the innate immune cell subsets, were associated with a significant increase in cytotoxic CD8 T cell infiltration with an activated phenotype, as shown by the expression of markers such as ki67, PD-1, T-bet and granzyme B. Unlike macrophage conversion, their depletion with clodronate does not affect CD8 T cell infiltration and activation and does not inhibit OS tumor growth, suggesting that it is better to reprogram the myeloid compartment rather than depleting macrophages “tout court” from the TME.

Gene expression analysis of the tumor lesions for selected immune-related genes, confirmed a clear induction of markers associated with T cell activation and recruitment in SD101-treated mice (*Perforin, Icos, Cd38, Ifng, Il2ra, Il7 and Cxcl10*). Interestingly, those changes in immune cell composition and activation state were observed in the TME of the SD101-injected and, partially, in the contralateral untreated lesions, supporting the hypothesis that its local administration is able to exert an abscopal effect, likely through the induction of a systemic immune response. As SD101 has been reported to induce systemic proinflammatory cytokine production, i.e. IL-1β and IP-10 [27], and we indeed confirmed the expression of these, and others, immune mediators in the TME of both treated and untreated contralateral lesions, we investigated whether the systemic anti-tumor effect observed in the contralateral, untreated lesion, was mediated by this “non-specific “ immune response or by the induction/activation of anti-tumor specific cytotoxic T cells by comparing SD101 therapeutic activity in immune-compentent and -deficient mice. Interestingly, while the tumor-inhibitory effect on the treated lesion was similar in the two strains, the efficacy in restraining the growth of the distant lesion in T cell-deficient animals was reduced and not statistically significant, suggesting the involvement of T cells in the induction of an abscopal effect. As further confirmation of this hypothesis, TCR sequencing analysis of tumor infiltrating CD8 T cells showed increased CD8^+^ T cell clonality both locally, in treated tumors, and sistemically, in untreated tumors. Remarkably, some of the clones found expanded in SD101-treated lesions were also present in the distant, untreated tumors, suggesting that tumor-specific CD8 T cells re-circulated systemically toward the secondary tumors.

As expected from the interferogenic activity of type C TLR9 agonists, we observed a substantial increase in the expression of PD-L1 on myeloid cells. In light of this finding and of several reports showing a higher therapeutic efficacy of TLR9 agonists when combined with immune checkpoint blocking antibodies [22, 26, 27], we tested whether this additive/synergistic effect occurs in our osteosarcoma models but we did not observe any difference in the therapeutic efficacy of SD101 administered alone or in combination with systemic anti PD-1 antibody, the latter also ineffective as monotherapy [16]. In contrast, we recently showed in the same OS models that the combination of anti PD-1 antibody with trabectedin, a marine-derived chemotherapeutic agent, enhanced the efficacy of the drug [16]. Also trabectedin induced TME reprogramming and increased the number of CD8 + T cells that expressed high level of PD-1. However, in the present study, CD8 tumor-infiltrating T cells, in addition to PD-1, expressed high level of other activation markers, such as T-bet, granzyme B and ki67 suggestive of their activated, not-exhausted, state, sufficient for the systemic anti-tumor immune response and therefore not in need of immune checkpoint blockade. The lack of additive/synergistic effects could then be attributed to the overwhelming activity of SD101. In contrast, in the experimental lung metastases model in which OS cells were injected intra-venously, SD101 administered by intranasal inhalation was largely unable to exert anti-tumor effects, unless combined with anti PD-1 antibody, an association capable of reducing both the number and size of lung metastases.

As OS preferentially disseminates into the lungs, the above approach could integrate the standard-of-care treatment regimen with the possibility of reducing chemotherapy drug dosages, which are often responsible for severe toxicities and long-term side effects. Additionally, while intralesional administration of TLR9 agonists in OS lesions grown in their physiological bone sites would be technically challenging in a clinical scenario, its administration by areosol in the lung metastatic setting, in combination with other systemic therapies, could be easily feasible.

The clinical application of intralesional administration of the SD101 TLR9 agonist has proven efficacy in untreated indolent lymphomas, in combination with local radiotherapy, achieving in some patients a reduction in tumor size not only of the treated lesion but also in their untreated sites [28]. A recent phase Ib trial assessed the safety, efficacy, and pharmacodynamics of SD101 in combination with pembrolizumab in patients with advanced melanoma and reported no major toxicities, an overall response rate (ORR) of 78% and a profound immune activation of the tumor microenviroment, with increased expression of Th1 cell-related genes and concomitant decrease in Th2 genes [29]. Additional clinical trials in other tumor histotpyes such as primary liver tumors (NCT05220722) and metastatic uveal melanoma are currently underway (NCT04935229).

## Conclusions

In conclusion our work demonstrates the anti-tumor activity of a TLR9 in OS, a high-grade, aggressive tumor for which no targeted therapies or efficient immunotherapies are available. This study provides evidence that reprogramming of the tumor immune microenvironment in a poorly infiltrated “cold” tumor type, such as OS, could represent a valuable way to increase the efficacy of standard or novel therapeutic approaches.

## Supplementary Information

**Additional file 1.** 

**Additional file 2.** 

**Additional file 3.** 

**Additional file 4.** 

**Additional file 5.** 

**Additional file 6.** 

## Data Availability

The raw data generated in this study are available upon request, from the corresponding author.

## Abbreviation

H&E: Hematoxylin and eosin
IF: Immunofluorescence
IHC: Immunohistochemistry
TAM: Tumor-associated macrophages
TLR9: Toll-like receptor 9
TMA: Tumor microenvironment
ORR: Overall response rate
OS: Osteosarcoma
RT-PCR: Real time polymerase chain reaction
TCR: T cell receptor
