# Supplementary material for: Rewiring innate and adaptive immunity with TLR9 agonist to treat osteosarcoma
Source: J Exp Clin Cancer Res. 2023 Jun 26;42:154. doi: 10.1186/s13046-023-02731-z (PMC10291774; doi:10.1186/s13046-023-02731-z)
Supplement: Supplementary file 2 — Additional file 2. [file 13046_2023_2731_MOESM2_ESM.docx]

**Additional file 2**


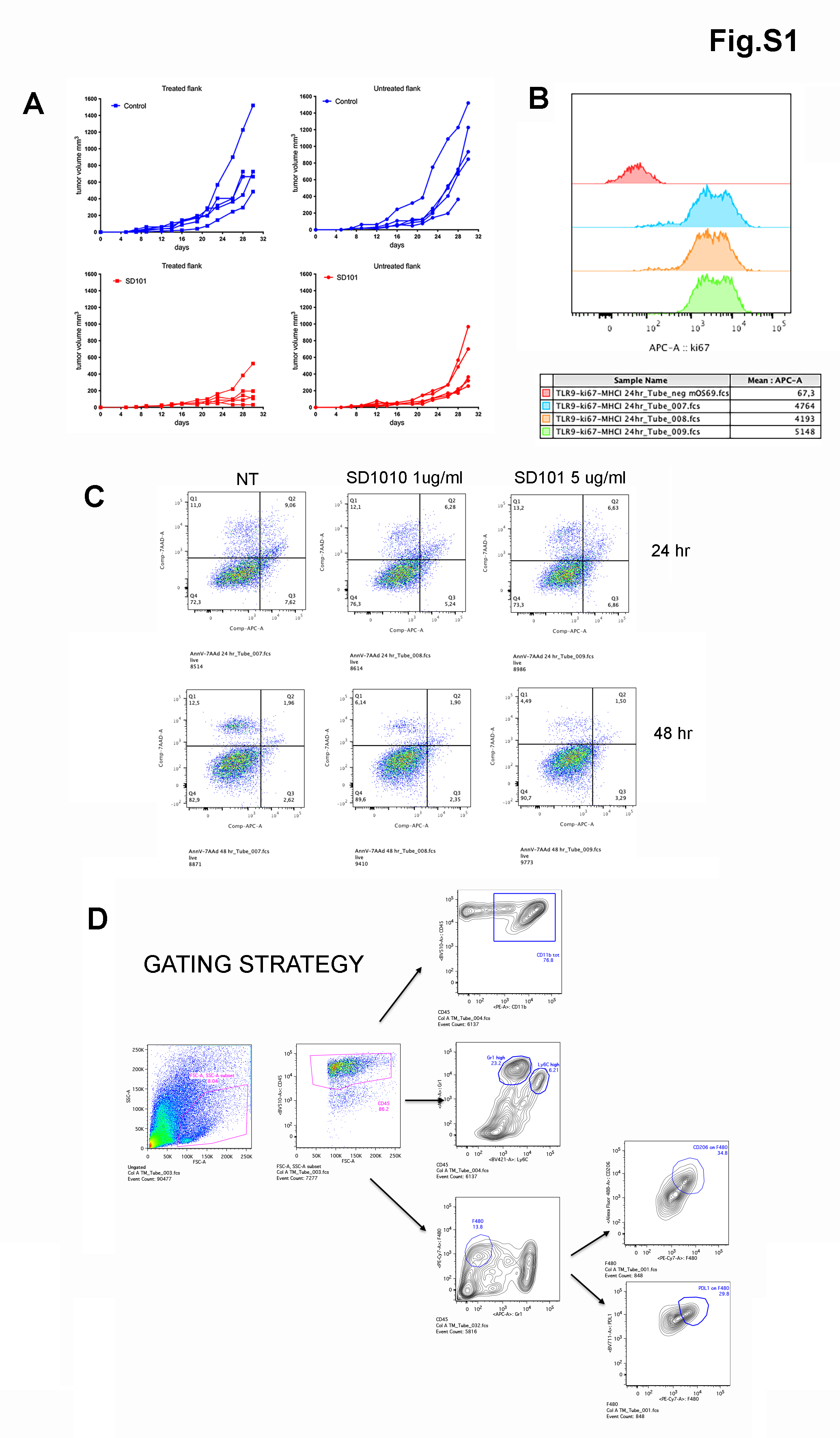


*Figure S1 Tumor volume for single mice, in vitro effect of SD101 and flow cytometry gating strategy.*

A. Graphs display tumor volume for each mouse from experiment shown in Figure 1. mOS69 cells were injected at the dose of 2x10^5^ cells on both flanks of the mice. SD101 (or saline as control) was injected intratumorally only in the left tumor lesion at the dose of 25 μg twice a week, for a total of 4 doses, starting when tumors reached 4–5 mm diameter. One graph is shown for each group of treatment. Left panels: treated flank, right panels: untreated flank. B. Tumor cells were treated in vitro for 24 and 48 hr with SD101 at the doses of 1 and 5 μg/ml. Proliferation was assessed by flow cytometry with intracelllar staining with ki67 antibody. C. Apoptosis was investigated by flow cytometry staining cells for AnnexinV and 7AAD to identify early apoptotic cells (AnnV+/7AAD-), late apoptotic cells (AnnV+/7AAD+), and necrotic cells (AnnV-/7AAD+). D. Gating strategy for multiparamentric flow cytometry analysis of myeloid cell subsets in tumor samples. Marker used are CD45 (total leukocytes), CD11b (total myeloid cells), Ly6G (granulocytic cells), Ly6C (monocytic cells), F4/80 (macrophages), CD206 on F4/80+ macrophages (M2-like macrophages), and PD-L1 on F4/80+ macrophages.
