# Supplementary material for: Rewiring innate and adaptive immunity with TLR9 agonist to treat osteosarcoma
Source: J Exp Clin Cancer Res. 2023 Jun 26;42:154. doi: 10.1186/s13046-023-02731-z (PMC10291774; doi:10.1186/s13046-023-02731-z)
Supplement: Supplementary file 3 — Additional file 3. [file 13046_2023_2731_MOESM3_ESM.docx]

**Additional file 3**

*
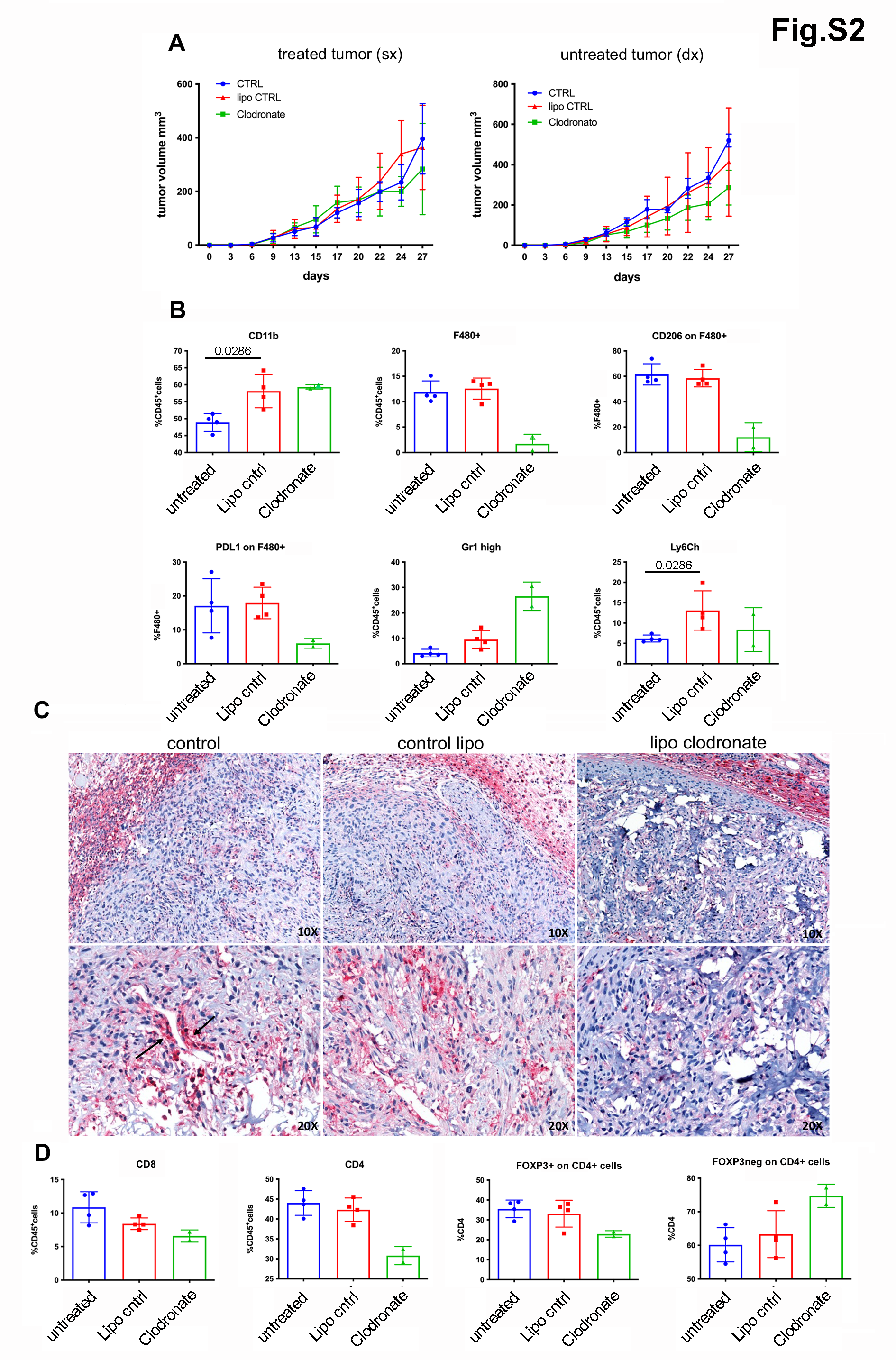
*

*Figure S2. Macrophage depletion does not affect mOS69 tumor growth.*

A. Tumor growth of mOS69 cells upon macrophage depletion. mOS69 cells were injected at the dose of 2x10^5^ cells on both flanks of the mice. For macrophage depletion, when tumors reached 4–5 mm diameter, mice were treated three times a week with Clodronate, control liposomes intra-tumor (50μl/mouse), or left untreated, for 3 weeks. Graphs show mean tumor volume for treated (left panel) and untreated (right panel) tumors. Five animals per group were used. B. Multiparametric flow cytometry analysis of tumor infiltrating myeloid cells in treated lesions from control , clodronate- or control liposomes-treated mice. Antibodies for CD11b (total myeloid cells), F4/80 (macrophages), CD206 (M2-like macrophages), Ly6G (granulocytic cells), Ly6C (monocytic cells) and PD-L1. Data are expressed as percentage of specific cell subsets (shown on the Y axis). Data from single mice are shown. Mann-Whitney test was used for statistical analysis. D. Representative immunohistochemistry staining of F4/80 on tumor lesions, to show effective intra-tumor macrophage depletion. Left panel: control lesion; middle panel: control liposomes-treated lesion; right panel: clodronated-treated lesion.Two different enlargments are shown. D. Multiparametric flow cytometry analysis of tumor infiltrating lymphoid cells in treated lesions from control, clodronate- or control liposomes-treated mice. Data for CD8, CD4, FOXP3+ (T regulatory cells) and FOXP3- (conventional CD4 T cells) are shown. Data are expressed as percentage of specific cell subsets (shown on the Y axis). Data from single mice are shown.
