# Supplementary material for: Rewiring innate and adaptive immunity with TLR9 agonist to treat osteosarcoma
Source: J Exp Clin Cancer Res. 2023 Jun 26;42:154. doi: 10.1186/s13046-023-02731-z (PMC10291774; doi:10.1186/s13046-023-02731-z)
Supplement: Supplementary file 4 — Additional file 4. [file 13046_2023_2731_MOESM4_ESM.docx]

**Additional file 4**


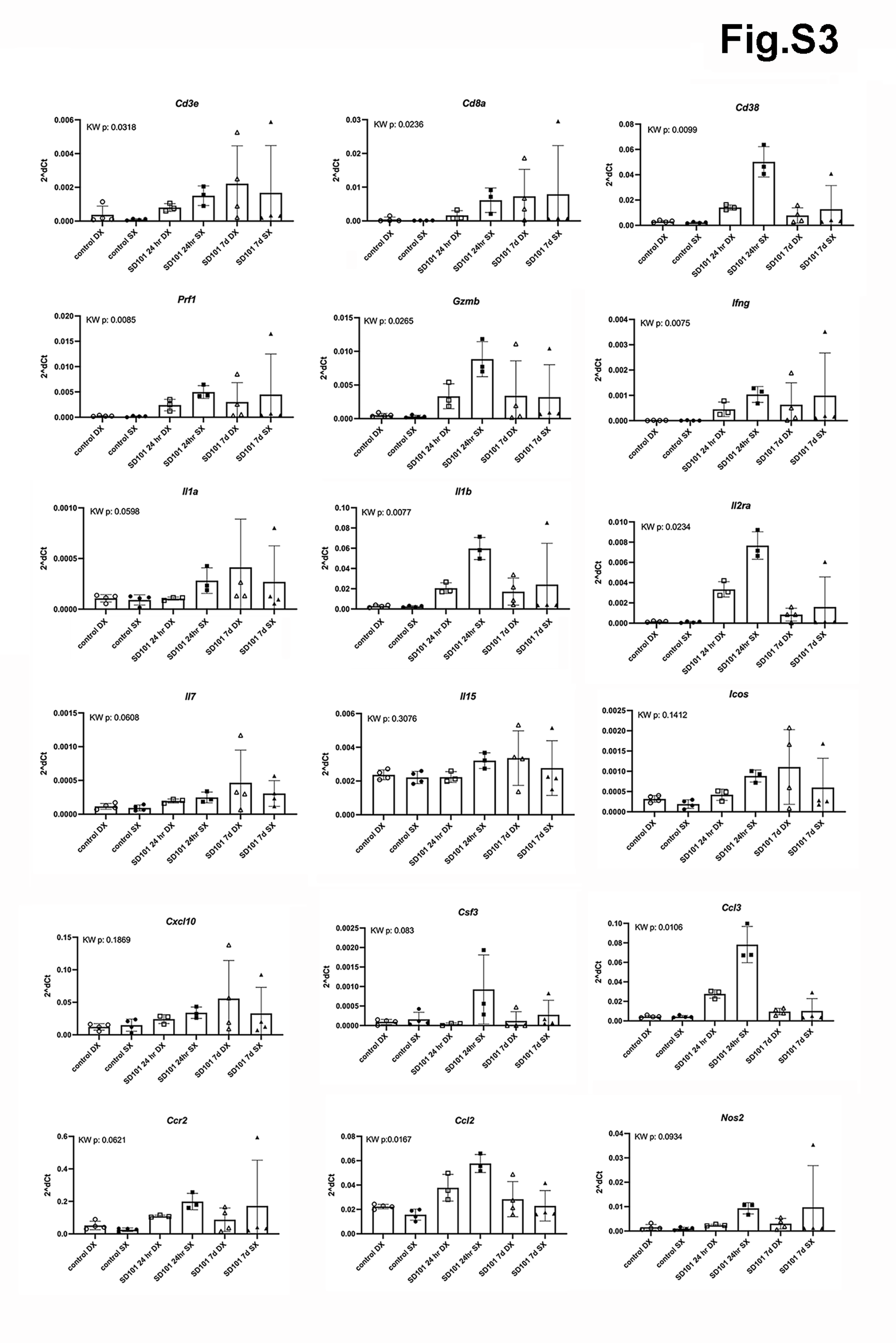


*Figure S3. Gene expression analysis of immune-related genes in SD101-treated mice.*

Gene expression analysis of several inflammation/immune-related markers using theTaqMan® array mouse immune panel in tumor samples from mice at 24 hours or 7 days after the last SD101 treatment.

The following genes are shown: *Cd8a, Cd3a, GrzmB*, *Cd38, Il2ra, Il7, Ifng, Prf1, Icos*, *Il1a and Il1b*, *Cxcl10*, *Ccl2 and 3, Ccr2, Csf3,* and *Nos2.* Both treated (left tumors, SX) and untreated (right tumors, DX) tumors were analyzed (3-4 samples for each time point and each group were analyzed). ANOVA was performed with Kruskal Wallis (KW) test for statistical analysis.
