# Supplementary material for: Rewiring innate and adaptive immunity with TLR9 agonist to treat osteosarcoma
Source: J Exp Clin Cancer Res. 2023 Jun 26;42:154. doi: 10.1186/s13046-023-02731-z (PMC10291774; doi:10.1186/s13046-023-02731-z)
Supplement: Supplementary file 5 — Additional file 5. [file 13046_2023_2731_MOESM5_ESM.docx]

**Additional file 5**


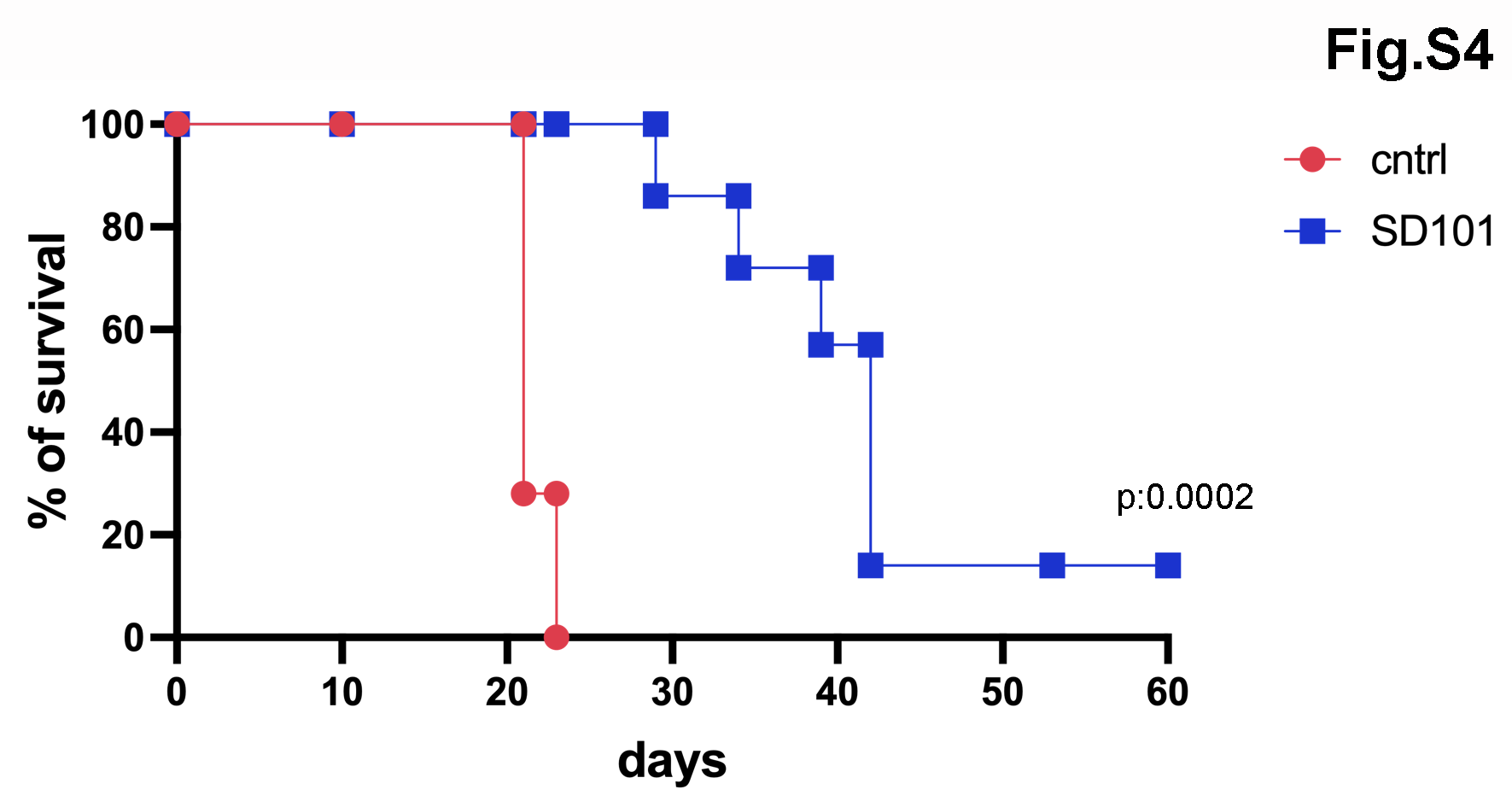


*Figure S4 Intralesional injection of SD101 extends the survival of treated mice.*

mOS69 cells were injected at the dose of 2x10^5^ cells on the left flank of the mice. SD101 (or saline as control) was injected intratumorally at the dose of 50 μg twice a week, for a total of 4 doses, starting when tumors reached 4–5 mm diameter. and then left untreated to assess the effect on survival. For ethical reason, animal human endpoint was set when tumors reached 10 mm diameter.

Seven animals were used in the each group. Log-rank test was used for statistical analysis.
