# Supplementary material for: Rewiring innate and adaptive immunity with TLR9 agonist to treat osteosarcoma
Source: J Exp Clin Cancer Res. 2023 Jun 26;42:154. doi: 10.1186/s13046-023-02731-z (PMC10291774; doi:10.1186/s13046-023-02731-z)
Supplement: Supplementary file 6 — Additional file 6. [file 13046_2023_2731_MOESM6_ESM.docx]

**Additional file 6**


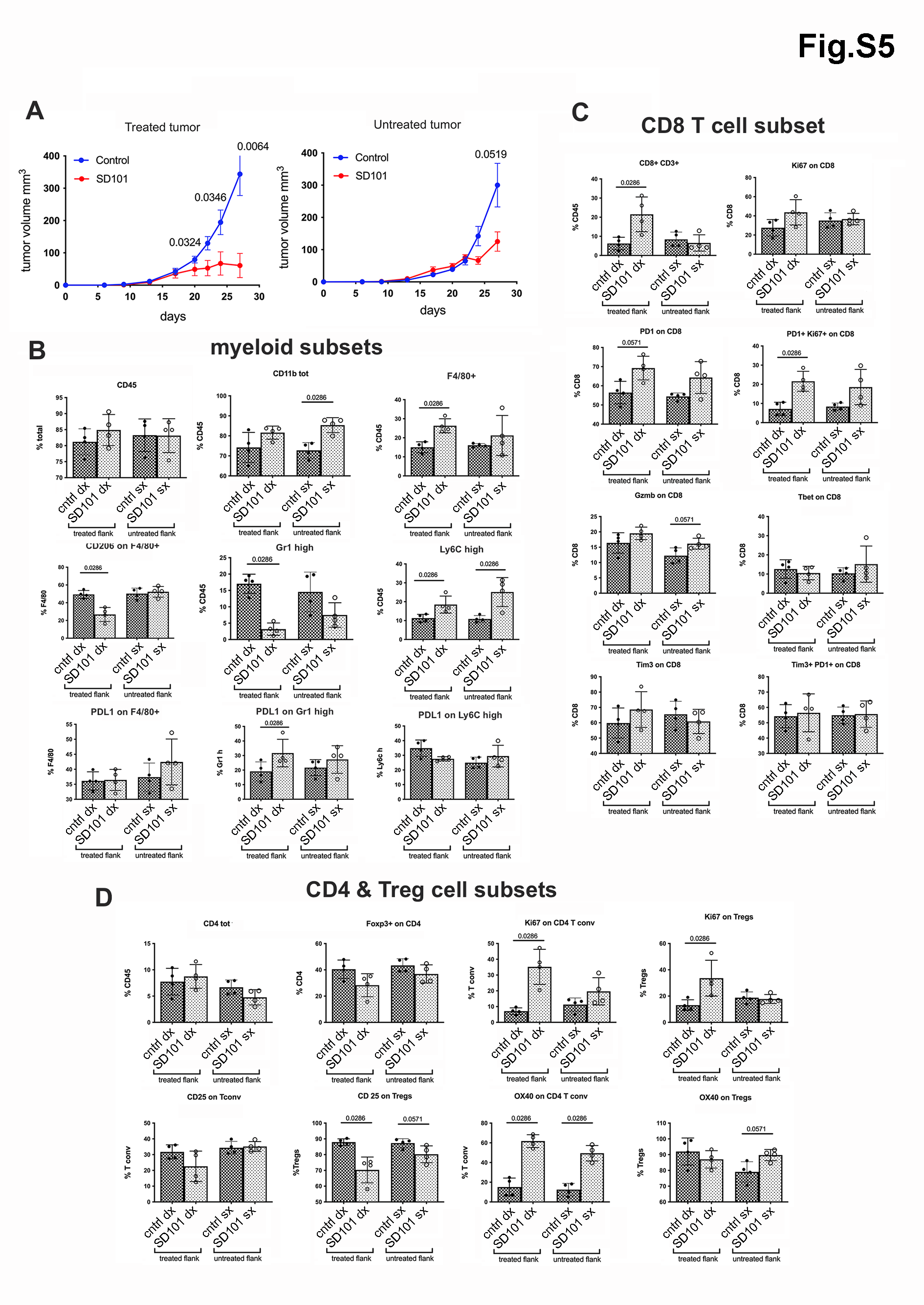


*Figure S5. Intralesional injection of SD101 is effective in K7M2 OS model.*

A. K7M2 cells were injected at the dose of 10^6^ cells on both flanks of the mice. SD101 (or saline as control) was injected intratumorally only in the left tumor lesion at the dose of μg twice a week, for a total of 4 doses, starting when tumors reached 4–5 mm diameter. Graphs show mean tumor volume for treated (left panel) and untreated (right panel) tumors. Six animals per group were used. Multiple unpaired t tests, one per time points, were used for statistical analysis. B. Multiparametric flow cytometry analysis of tumor infiltrating myeloid cells in both treated and untreated lesions from SD101- or PBS-treated mice. Antibodies for CD45 (total leukocytes), CD11b (total myeloid cells), CD11c (dendritic cells), F4/80 (macrophages), CD206 (M2-like macrophages), Ly6G (granulocytic cells), Ly6C (monocytic cells) and PD-L1. C. Multiparametric flow cytometry analysis of tumor infiltrating CD8 T cells in both treated and untreated lesions from SD101- or PBS-treated mice. Antibodies for CD3, CD8, ki67, granzyme B, Tbet, PD-1 and TIM3 were used to assess activation, exhaustion and proliferation. D. Multiparametric flow cytometry analysis of tumor infiltrating CD4 T cells in both treated and untreated lesions from SD101- or PBS-treated mice. Antibodies for CD3, CD4, FOXP3 (for T regulatory cells), ki67, CD25 and OX40 were used to identify T regulatory cells (Tregs) and conventional (Tconv) CD4 T cells, and their activation and proliferation. For all flow cytometry analysis data from single mice are shown (4 mice per group). Mann-Whitney test was used for statistical analysis.
